# The Risk Factors of Acute Coronary Syndrome in Young Women: A Systematic Review and Meta-Analysis

**DOI:** 10.2174/1573403X19666221116113208

**Published:** 2023-03-22

**Authors:** Sisca Natalia Siagian, Christianto Christianto, Phoniex Angellia, Ho Indra Holiyono

**Affiliations:** 1 Department of Cardiology and Vascular Medicine, Pediatric Cardiology and Congenital Heart Defect Division, National Cardiovascular Center Harapan Kita, Universitas Indonesia, Jakarta, Indonesia;; 2 Faculty of Medicine, Universitas Indonesia, Jakarta, Indonesia

**Keywords:** Acute coronary syndrome, atherosclerosis, meta-analysis, risk factors, ACS, young women

## Abstract

**Background:**

Acute coronary syndrome (ACS) has been one of the leading causes of mortality in the world. Despite common understanding regarding ACS as an older population’s or man's disease, the number of young women affected by this condition is increasing. Many studies have assessed the risk factors of ACS, but only a few studies focused on this subpopulation. Therefore, this systematic review and meta-analysis aim to evaluate the risk factors predisposing to ACS in the young women population.

**Methods:**

Nine online databases were screened from the date of inception to September 2021, where the acquired studies were evaluated using the PRISMA statement. The inclusion criteria were a case control study with women age cut-off of <50 years. The risk factors of acute coronary syndrome were analyzed using a random-effect model, expressed as summary statistics of odds ratio (OR) for categorical variable and standard mean difference (SMD) for continuous data with normal distribution, with 95% confidence interval (CI). Quality assessment was conducted using the STROBE statement.

**Results:**

Seven studies with the total of 7042 patients met the inclusion criteria of this meta-analysis. Diabetes mellitus, high BMI, obesity, hypercholestrolemia, hypertension, smoking, and family history significantly increased acute coronary syndrome risk in young women. Other risks such as heavy alcohol consumption, oral contraceptive use, and postmenopausal state were associated with higher risk of ACS.

**Conclusion:**

The independent risk factors which are strongly related to ACS in young women were diabetes mellitus, hypertension, and hypercholesterolemia with odd ratios of 6.21, 5.32, and 4.07. Other risk factors which may be associated with an increased risk of ACS in young women were heavy alcohol consumption, oral contraceptive use, and postmenopausal state. Health promotion and effective intervention on this specific population regarding these risk factors can decrease young female cardiovascular morbidity and mortality as well as improved quality of life of women.

## 1 INTRODUCTION

Acute coronary syndrome (ACS) has been one of the leading causes of mortality in the world [1]. Despite common understanding regarding ACS as an older population’s or man's disease, the number of young populations, particularly women, affected by this condition is increasing [2, 3]. The prevalence of a cardiovascular disease among women was estimated to be 275.2 million cases worldwide in 2019 [4].

MI hospitalization rates in women aged 35-44 were found to be increased [3], where they had worse clinical outcomes than men of similar age with a 30-day mortality of 6.2% [5, 6]. Many studies have assessed the risk factors of ACS, but only a few studies focused on subpopulations such as young women [7]. Even though a few independent studies had observed the predisposing factors of ACS in this specific group, there was no study that analyzed nor combined their findings to estimate the overall effects. Therefore, the aim of this systematic review and meta-analysis was to evaluate the risk factors predisposing to ACS in the young women population.

## 2 MATERIALS AND METHODS

### 2.1 Search Strategy

**Fig. (1):**
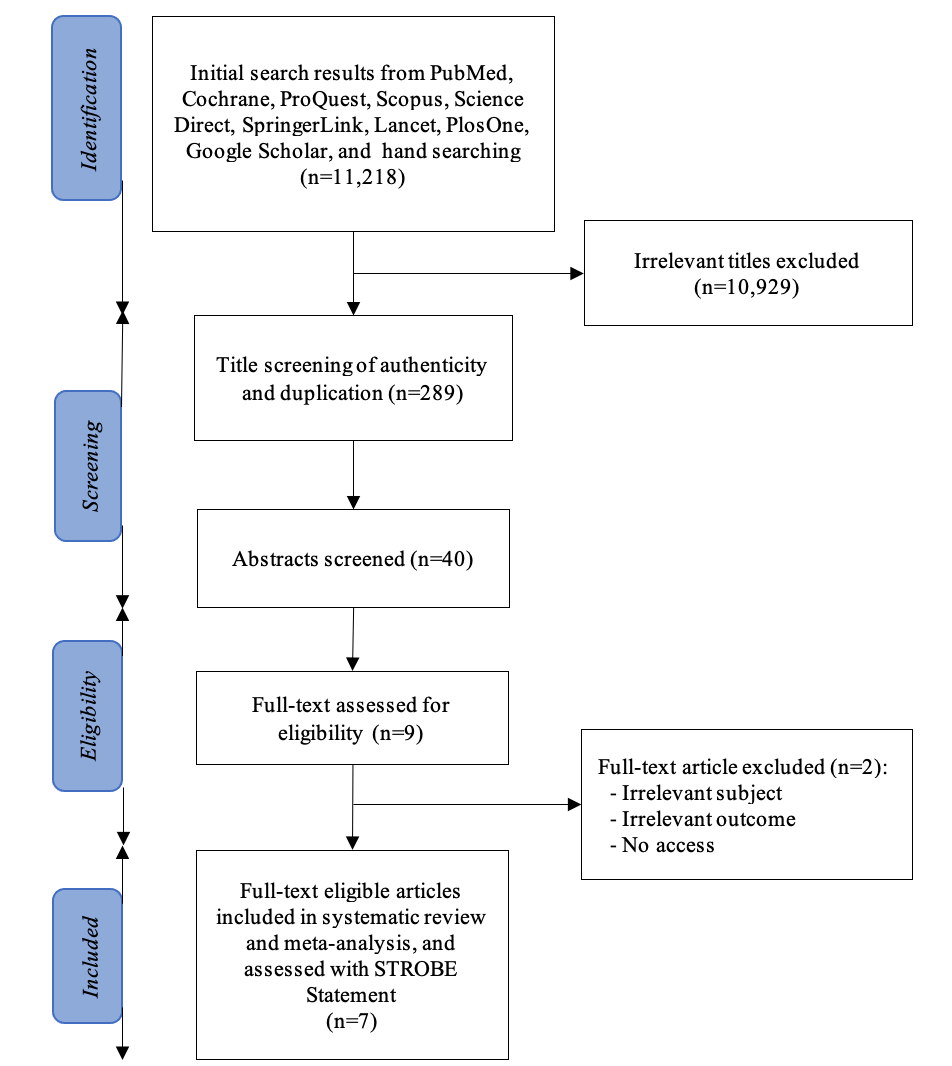
PRISMA flow of the systematic review and meta-analysis [8].

This meta-analysis was conducted in accordance with the Preferred Reporting Items for Systematic Review and Meta-Analysis (PRISMA) statement (Fig. **1**) [8]. A systematic search in PubMed, Scopus, Cochrane, Springerlink, ProQuest, ScienceDirect, Lancet, PlosOne, and Google Scholar databases was performed using the combination of keywords (acute coronary syndrome OR acute myocardial infarct AND young AND woman AND risk factor). AND (young) AND (woman) AND (risk factor). The database search and hand searching were done independently in September 2021 by three reviewers (C, IH, PA) with equal participation.

### 2.2 Study Criteria

Several eligibility criteria were defined for the included studies. The inclusion criteria were case control studies that assessed risk factors of acute coronary syndrome in the young women population with an age cut-off of <50 years old. The cases were young women diagnosed with acute coronary syndrome (ACS), and the controls were young women without ACS (including any previous ACS). The exclusion criteria were studies which compared young women with other populations (young men, older women, *etc*.), studies with ambiguous age cut-off, studies in the form of case reports, case series, editorials, reviews, or meta-analyses, and studies with irretrievable full-text articles.

### 2.3 Data Extraction and Quality Assessment

**Fig. (2):**
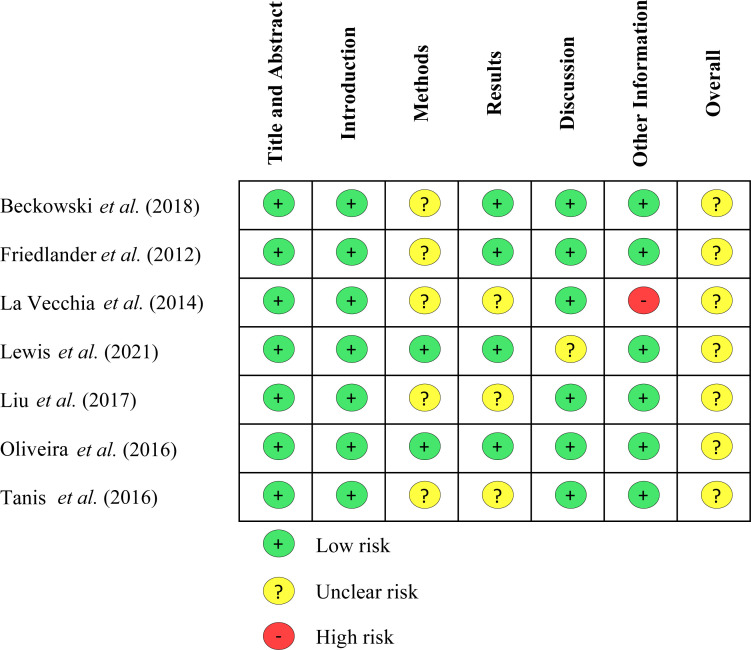
Quality appraisal using STROBE statement [9].

The screening and data extraction of the included studies were accomplished by three reviewers (C, IH, PA). The data extracted from the included studies were study and patient characteristics (first author, year of publication, study design, settings, study interval, type of ACS, young woman age cut-off, risk factors assessed, in addition to number and age of the patients in both case and control arm), the risk factors in either case or control group (obesity, hypercholesterolemia, hypertension, diabetes mellitus, smoking, family history of coronary artery disease, and other risk factors: race, high alcohol use, coffee consumption, oral contraceptive use, parity, marital status, and post-menopausal state). Quality assessment of the included studies was performed by three reviewers (C, IH, PA,) using the Strengthening the Reporting of Observational Studies in Epidemiology (STROBE) statement [9]. Any difference of opinion in the data extraction or the quality assessment was settled by discussion between the three reviewers to reach an agreement (Fig. **2**).

### 2.4 Statistical Analysis

Parametric data are shown as mean ± standard deviation (SD), while non-parametric data are shown as median (interquartile range). The risk factors of acute coronary syndrome were analyzed using a random-effect model, expressed as summary statistics of odds ratio (OR) for categorical variables and standard mean difference (SMD) for continuous data with normal distribution, with 95% confidence interval (CI). A statistically significant hypothesis was confirmed by a p-value of <0.05. Heterogeneity between studies was assessed using I2 statistics. All statistical analyses were done using REVMAN (version 5.4; Cochrane Collaboration, Oxford, UK) [10].

## 3 RESULTS

### 3.1 Search Results

The PRISMA flow diagram of the literature selection for this systematic review and meta-analysis is shown in Fig. (**1**). The initial search yielded 11,218 potential studies from the selected databases. The exclusion of studies with irrelevant titles generated 289 studies for authenticity and duplication screening. Forty studies were qualified for abstract assessment, eliciting 9 studies for full-text evaluation. Two studies were eliminated due to irrelevant subjects, inapplicable outcomes, and no access to full-text papers. Conclusively, seven studies complied with the eligibility criteria and thus were included in this systematic review and meta-analysis.

### 3.2 Study Characteristics

This systematic review and meta-analysis cover 7 case-control studies that were published from 1987 to 2020 in Poland, USA, Italy, Germany, UK, France, Austria, Switzerland, China, Portugal, and Netherlands. The total case participants were 2956 patients, while the control ones were 4086 patients [11-17]. Unstable angina (UA), non-ST-segmen elevation myocardial infarction (NSTEMI), or ST-segmen elevation myocardial infarction (STEMI) were reported in 3 studies [11, 15, 16]. Unfortunately, the remaining 4 studies did not detail the diagnoses of acute myocardial infarction [12-14, 17]. Each study applied a different cut-off for age categorization with 4 studies <45 years old, 2 studies <46 years old, and one study <50 years old [11-17]. In general, the control group was younger than the case group with the mean age ranging from 35.0 ± 7.7 to 40.57 ± 4.01 years and 39.5 ± 4.5 to 42.8 ± 6.1 years, respectively [12, 15-17]. There was one study that presented age as a median of 42 (39-44) years for both arms [11]. However, 2 studies did not specify the age of patients [13, 14]. Despite various risk factors observed in Table **1**, this study only focused on the major ones, such as BMI and obesity, hypercholesterolemia, hypertension, diabetes mellitus, smoking, and family history of CAD. Furthermore, we also reviewed other risk factors, including alcohol intake, coffee consumption, oral contraceptive, parity, marital status, and post-menopause.

### 3.3 Diabetes Mellitus

**Fig. (3):**
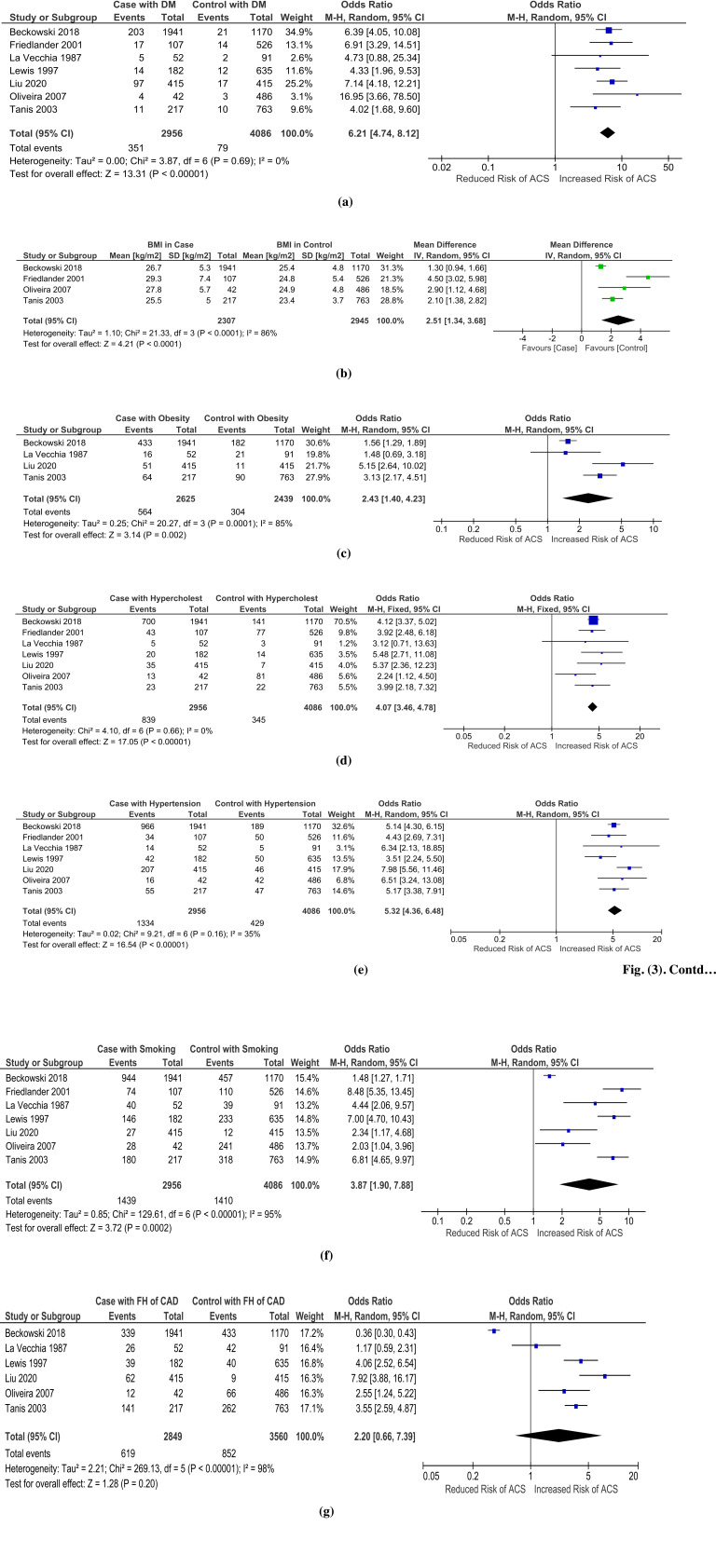
Forest plot of risk factors of ACS in young women: (**a**) Diabetes Mellitus, (**b**) BMI, (**c**) Obesity, (**d**) Hypercholesterolemia, (**e**) Hypertension, (**f**) Smoking, (**g**) Family history of coronary artery disease.

All included studies revealed increased risk of ACS in population of women with diabetes mellitus (7 studies, Odds ratio, 6.21 95% CI 4.74 - 8.12; P < 0.00001; I^2^ = 0%) (Fig. **3a**). Diabetes mellitus was found significantly higher in ACS case population (5%-23%) in comparison with control patients (1%-4%) (Table **2**) [11-17].

### 3.4 Body Mass Index and Obesity

The BMI of the case population was higher than the control population in all studies (4 studies, Odds ratio, 2.51 95% CI 1.34 - 3.68; P < 0.00001; I^2^= 86%) (Fig. **3b**) [11, 12, 16, 17]. This finding is consistent with the incidence of obesity in available studies, which showed an increased occurrence of obesity in case population (4 studies, Odds ratio, 2.43 95% CI 1.40 - 4.23; P < 0.00001; I^2^ = 85%) (Fig. **3c**) [11, 13, 15, 17]. Analysis of studies by Beckowski *et al.* (2018), La Vecchia *et al.* (1987), Liu *et al.* (2020), and Tanis *et al.* (2003) revealed an obesity odds ratio of 1.56 (1.29, 1.89), 1.48 (0.69, 3.18), 5.15 (2.64, 10.02), 3.13 (2.17, 4.51), respectively (Table **2**) [11, 13, 15, 17]. Nevertheless, the obesity cut-off was found to be different in available studies. Beckowski *et al.* (2018) and La Vecchia *et al.* (1987) used ≥30 kg/m2 and >25 kg/m2 as obese BMI cut-offs, while Liu *et al.* (2020) and Tanis *et al.* (2003) applied ≥24 and ≥27.3 kg/m2 as overweight or obese BMI cut-offs [11, 13, 15, 17].

### 3.5 Hypercholesterolemia

All included studies showed that hypercholesterolemia elevated the risk of ACS in young women (7 studies, Odds ratio, 4.07 95% CI 3.46 - 4.78; P < 0.00001; I^2^ = 0%) (Fig. **3d**) [11-17]. Lewis *et al.* (1997), Liu *et al.* (2020), and Beckowski *et al.* (2018) were 3 studies that portrayed the most prominent effect of hypercholesterolemia with an odds ratio of 5.48 (2.71, 11.08), 5.37 (2.36, 12.23), 4.12 (3.37, 5.02), respectively (Table **2**) [11, 14, 15]. The likelihood of getting ACS was markedly increased if hypercholesterolemia occurred in patients (case population 8.4% - 40.2% *vs* control population 1.7% - 16.7%) [11-17].

### 3.6 Hypertension

Hypertension consistently raised the probability of young women experiencing ACS (7 studies, Odds ratio, 5.32 95% CI 4.36 - 6.48; P < 0.00001; I^2^ = 35%) (Fig. **3e**) [11-17]. The three most significant associations between those variables were demonstrated by Liu *et al.* (2020), Oliveira *et al.* (2007), and La Vecchia *et al.* (1987) with an odds ratio of 7.98 (5.56, 11.46), 6.51 (3.24, 13.08), and 6.34 (2.13, 18.85), respectively (Table **2**) [13, 15, 16]. In all included studies, hypertension augmented the occurrence of ACS in the case population (23% - 49.8%) rather than in the control population (5.5% - 16.2%) [11-17].

### 3.7 Smoking

Young women who smoked either actively or passively were prone to suffer ACS in their life (7 studies, Odds ratio, 3.87 95% CI 1.90 - 7.88; P < 0.00001; I^2^ = 95%) (Fig. **3f**) [11-17]. Studies conducted by Friedlander *et al.* (2001), Lewis *et al.* (1997), and Tanis *et al.* (2003) reported the three greatest correlations between smoking and ACS with an odds ratio of 8.48 (5.35, 13.45), 7.00 (4.70, 10.43), and 6.81 (4.65, 9.97), respectively (Table **2**) [12, 14, 17]. All included studies agreed that ACS incidence was elevated if young women had smoking habits (case population 6.5% - 82.9% *vs* control population 2.9% - 49.6%) [11-17].

### 3.8 Family History of Coronary Artery Disease

Most studies showed a positive correlation between family history and increased risk of ACS in young women (6 studies, Odds ratio, 2.20 95% CI 0.66 - 7.39; P < 0.00001; I^2^ = 98%) (Fig. **3g**) [11, 13-17]. Studies by Lewis *et al.* (1997), Liu *et al.* (2020), Oliveira *et al.* (2007), and Tanis *et al.* (2003) demonstrated a significant increase in the risk of ACS for women with CAD family history, with an odds ratio of 4.06 (2.52, 6.54), 7.92 (3.88, 16.17), 2.55 (1.24, 5.22), and 3.55 (2.59, 4.87), respectively (Table **2**) [14-17]. La Vecchia *et al.* (1987) reported a small decrease in family history incidence, by 50% in the case arm compared with 46.2% occurrence in the control arm [13]. However, Beckowski *et al.* (2018) revealed an inversed correlation between family history and ACS with an odds ratio of 0.36 (0.30, 0.43) [11].

### 3.9 Other Risk Factors

Studies by Friedlander *et al.* (2001) and Tanis (2003) showed an insignificant proportion of the Caucasian race between the case and control group, with a ratio of 85% *vs* 87.6% and 95% *vs* 94%, respectively (Table **2**) [12, 17]. Alcohol intake of the ACS population was found to be higher in two studies [13, 16], where La Vecchia *et al.* (1987) found a moderate alcohol intake (<3 drinks/day) ratio of 37% *vs* 51% in the case *vs* control group, and 19% *vs* 7% ratio of heavy alcohol intake (≥3 drinks/day) in case *vs* control group, respectively [13]. However, a study by Lewis *et al.* (1997) reported a slight increase in alcohol intake in control patients compared with the case group (24% *vs* 19%) [14]. Coffee or caffeine consumption in the case group was higher in Friedlander *et al.* (2001), Oliveira *et al.* (2007), and La Vecchia *et al.* (1987) [12, 13, 16].

The number of married women was found to be slightly higher in the case group in a study by La Vecchia *et al.* (1987) (69% *vs* 67%) [13], while Friedlander *et al.* (2001) reported otherwise (65% *vs* 73%) [12]. Furthermore, La Vecchia *et al.* (1987) and Lewis *et al.* (1997) revealed a higher number of parity in young women with ACS with a ratio of 79% *vs* 67% and 92% *vs* 83%, respectively [13, 14]. La Vecchia *et al.* (1987), Lewis *et al.* (1997), Liu *et al.* (2020), and Tanis *et al.* (2003) showed higher use of oral contraceptives in case patients (4%-39% *vs* 0.4%-36%) [13-15, 17]. Two studies discovered a higher incidence of post-menopausal patients in the case group with a ratio of 4% *vs* 0.9% in the Liu *et al.* (2020) study and 13% *vs* 8% in the La Vecchia *et al.* (1987) study [13, 15].

## 4 DISCUSSION

ACS has been regarded as a man’s disease for centuries, and thus, there were many underdiagnosed and untreated ACS in women [3]. There was a general belief that women, especially those who were younger, rarely suffered from ACS, and those who had ACS were in exceptional circumstances [1]. While it is true that ACS mainly occurs in individuals >50 years; younger adults can be affected as well [5]. The incidence of ACS in women, including young women, was rising over the years, and ACS has been recognized as the biggest killer of women [1, 3]. According to previous studies, young women have significantly higher mortality and poorer prognosis compared to other women age groups and men. There were some hypotheses regarding this outcome, but some believed it was likely to be multifactorial [18].

The major risk factors associated with ACS were hypertension, hyperlipidemia, smoking, diabetes, and obesity [11]. Women usually have fewer risk factors for ACS compared to men. However, most studies showed that young women with ACS have significantly greater comorbidities compared to young men [3]. There were some studies that showed that the prevalence of risk factors for ACS in young women were different to other age groups and gender [11, 15, 16].

### 4.1 Diabetes Mellitus

The global prevalence of diabetes is estimated to increase continuously until 2030 due to aging, increasing prevalence of physical inactivity and obesity, and urbanization [19]. Individuals with diabetes have an increased risk for extensive CAD than nondiabetic individuals [20]. Diabetes promotes ACS through several mechanisms, such as hyperglycemia-induced oxidative stress, endothelial dysfunction, plaque disturbance, platelet activation, and alteration of coagulation [21, 22]. The interaction of these factors favors thrombus formation and generates proinflammatory state, resulting in a greater risk of atherosclerotic plaque rupture [21]. Although women have physiological estrogen protective effect from ACS risk, this effect is diminished in the diabetic population [23].

Menke *et al.* (2015) reported an increase in the incidence of diabetes in young women in the US population [24]. A meta-analysis concluded that diabetes affects ACS risk in women more than in men population [23]. Furthermore, a study by Franklin *et al.* (2004) stated that diabetic patients with ACS were more often women and more likely to have additional comorbidities [25]. Younger women were more likely to present with a history of diabetes, hypertension, and stroke in comparison to younger men [26, 27]. Moreover, the ACS mortality of diabetic women was found to be higher than men [23].

Our analysis resulted in an odds ratio of 6.21 (4.74 - 8.12) in the young women population. As a comparison, a study by Ricci *et al.* (2017) showed an odds ratio of 1.07 (0.98-1.17) [5], while Levit *et al.* (2011) reported an odds ratio of 4.26 (3.51-5.18) in general women population [28]. Kawano *et al.* (2006) also declared diabetes as an independent risk factor of MI in women with an odds ratio of 6.12 (3.78-12.02) [29].

### 4.2 BMI and Obesity

BMI is an easily attainable measure that is widely used as a parameter of obesity [30]. Obesity is most commonly defined as a BMI of ≥30 kg/m2 in the adult population [31, 32]. Nowadays, obesity prevalence follows an escalation trend, and 20% of world’s adults are estimated to be obese by 2030 [32, 33]. BMI and obesity are greatly associated with an increased risk of ACS [31, 32]. Studies have shown the risk’s relation to elevated blood lipids, blood glucose, and blood pressure [34]. Obesity influences fibrinolytic activity, increases cardiac workload, and alters lipid and glucose metabolism [35, 36].

Studies by Bucholz *et al.* (2017) and Choi *et al.* (2014) stated that women had more risk factors for cardiovascular disease, including obesity, compared to men [37, 38]. Davis *et al.* (2015) pointed obesity as a significant ACS risk factor in young women [7]. These statements are in accordance with our findings, where BMI and obesity achieved odds ratio of 2.51 (1.34 - 3.68) and 2.43 (1.40 - 4.23), respectively. However, two studies stated an insignificant association between obesity and ACS in young women, with odds ratio of 0.99 in study by Beckowski *et al.* (2018) and relative risk of 1.29 in study by La Vecchia *et al.* (1987) [11, 13].

### 4.3 Hypercholesterolemia

Several studies showed the increased risk of ACS in hypercholesterolemia patients [39, 40]. Particularly, it had the highest association with non-HDL cholesterol [41]. Cholesterol collection causes endothelial dysfunction, thus resulting in increased adhesion molecules, pro-inflammatory cytokines production, lowering of nitric oxide quantity, and recently involving NLRP3 inflammasome activation [42]. In young women with high LDL levels (≥ 186 mg/dL), one of the contributing factors involved molecular defined familial hypercholesterolemia with most mutations taking place in LDLR (90%) and a few in APOB gene (90%). In addition, unfavorable lifestyles were significantly associated with hypercholesterolemia in young women without those genetic defects [43]. In the present study, we found a positive correlation between hypercholesterolemia and ACS in young women with an odds ratio of 4.07 (3.46 - 4.78). It was in accordance with other studies that reported hypercholesterolemia in 67.5% - 71% of case patients [44, 45]. In contrast, Oda *et al.* (2013) and Kawano *et al.* (2006) did not find any significant association of those variables with an odds ratio of 1.00 (0.14 - 7.28) among women in the general population [29, 46].

### 4.4 Smoking

Smoking was thought to be one of the ACS major risk factors in many studies [47, 48]. Either active or passive smoking promotes the activation of endogenous sources of free radicals; activation of neutrophils, monocytes, platelets, and T cells; and the release of free radicals directly from cigarette smoke components. These mechanisms lead to decrease NO generation and create an oxidative stress state for the initiation and progression of the atherothrombotic disease [49]. Additionally, the association between smoking and ACS risk was found to be carried out by lipid mediation effect [50]. According to the PROSPECT trial, there were more women <65 years who reported a history of smoking than men (70.5% *vs* 56.6%) [51]. Mortality was elevated in women who smoked <15 cigarettes daily and ≥ 15 cigarettes daily with a hazard ratio of 1.99 (1.47 - 2.27) and 2.81 (2.47 - 3.20) [52]. In our study, we revealed an odds ratio of 3.87(1.90 - 7.88) for smoking as ACS risk factor. This value was in between the odds ratio of 2.64 (1.39 - 5.02) and 6.45 (1.48 - 28.57) that were presented by the other 2 studies [53, 54].

### 4.5 Hypertension

In many studies, hypertension was strongly linked to patients who were diagnosed with ACS [55, 56]. A complex interplay involving genetic predisposition, sympathetic hyperactivity, abnormal vasoactive circulating substance, and insulin resistance was recognized to be the pathogenesis of hypertension. Eventually, this interaction leads to endothelial dysfunction, increased permeability of intima, mechanical stress, and LVH promoting atherosclerosis, spasm, and plaque rupture [57]. PROMETHEUS multicenter US registry reported that women presenting ACS below 55 years were found to have higher frequencies of hypertension than men [58]. It was consistent with the GENESIS-PRAXY cohort study that traditional risk factors such as hypertension were more prevalent in women 40-49 years old than in men of similar ages (55% *vs* 43%) [38]. In addition, several studies also shared the same result, which was statistically significant [59, 60], especially with stage 1 diastolic hypertension [61]. In our study, hypertension was found to increase the risk of ACS in young women with an odds ratio of 5.32 (4.36 - 6.48). This value is higher compared with the odds ratio of 3.39 (1.16 - 3.54) and 1.66 (1.34 - 2.06) by other studies that used a cut-off of <55 years old [62, 63].

### 4.6 Family History

Several retrospective and prospective studies have investigated the association between family history and acute coronary disease incidence [64-67]. A study by Perkins *et al.* (1986) stated that increased risk of CAD related to positive family history was mediated by familial aggregation of major risk factors [68], while Hopkins *et al.* (1988) analyzed family history as an independent risk factor [69]. Family history of CAD was found to be higher in the younger population, where young women have a slightly higher proportion of ACS family history than young men. However, the odds ratio of family history in the women group was not significant with an OR of 1.01 (0.96-1.05) [5]. Our study found a significant ACS risk increase in young women with positive family history, with an odds ratio of 2.20 (0.66 - 7.39). This finding is relevant to the analysis by Colditz *et al.* (1986), where the relative risk for the young women population with parental history achieved 2.8 (2.0-4.1) [65].

### 4.7 Other Risk Factors

#### 4.7.1 Race

From our review, two studies showed that there is no significant difference in the proportion of the Caucasian race between case and control groups [12, 17]. Despite this insignificant result, studies showed that ethnically diverse women present with ACS at a younger age than Caucasian women. Black women have a higher prevalence of myocardial infarction compared to all other racial and ethnic groups of women [70]. Black women and Hispanic white women usually present with more comorbidities (diabetes, hypertension, heart failure, obesity), and experience longer delays before treatment with worse outcomes compared to non-Hispanic white patients [71]. Studies also showed that black and Hispanic ACS patients were more likely to be younger and female. The relationship between races and ethnicities with ACS in young women is very complex, and further studies are required to investigate this [70, 71].

#### 4.7.2 Alcohol

From what we had known, heavy alcohol consumption is associated with an increased risk for hypertension and, thus, a major risk factor for coronary heart disease. However, moderate alcohol intake has a beneficial effect in reducing the risk of ACS, which is believed to be due to increased levels of high-density lipoprotein cholesterol, increased insulin sensitivity, and decreased fibrinogen [72]. This was consistent with the study by La Vecchia *et al.* (1987), which showed that more proportion of young women with ACS had heavy alcohol intake compared to the control group, while moderate alcohol intake seemed to protect young women from ACS [13]. Other studies did not specify the amount of alcohol intake, thus causing different results. Therefore, there is a dose-response relationship between alcohol intake and the risk factor for ACS in young women [13, 72]

#### 4.7.3 Caffeine

In the included studies, coffee or caffeine consumption was higher in young women who had ACS compared to the control group [12, 13, 16]. La Vecchia *et al.* (1987) reported that age-adjusted risk estimates were elevated only for heavy coffee drinkers (more than four cups per day). However, the finding in this study may be explained by the high correlation between smoking and coffee consumption with the multivariate relative risk being insignificant [13]. Willett *et al.* (1996), who conducted a ten-year follow-up study, also found that coffee consumption, regardless of the amount of coffee, was not a risk factor for ACS in women [73]. A dose-response meta-analysis of 17 observational studies also reported no association between coffee consumption and ACS in women [74].

#### 4.7.4 Marital Status and Parity

There were inconsistent results between the included studies, in addition to the insignificant differences between case and control groups regarding marital status and the risk of ACS in young women [12, 13]. La Vecchia *et al.* (1987) reported that young women with one or two births had an insignificant elevated risk for ACS compared to nullipara women, in contrast to women with three or more births who had a slightly reduced risk for ACS [13]. Although most studies reported increased risk for ACS in young women who had their first pregnancy at an early age (less than 20-25 years), the relative risk was not high. Therefore, early pregnancy and parity is only a minor contributing risk factor for ACS in young women [75, 76].

#### 4.7.5 Oral Contraceptive

Oral contraceptives, especially combined oral contraceptives, are known to cause venous as well as arterial thrombosis, but the association of oral contraceptives with arterial thrombosis, such as myocardial infarction, is still not well-established [77]. Arterial thrombosis is less likely to occur with the use of oral contraceptives in the absence of cardiovascular risk factors. However, a few studies reported that oral contraceptives increased the risk of ACS by two folds in reproductive-aged women [78, 79]. A meta-analysis study also reported a 1.6-fold increased risk of myocardial infarction in women using combined oral contraceptives [80]. The included studies also reported consistent results in which there were more oral contraceptive users in young women with ACS [13-15, 17].

#### 4.7.6 Postmenopausal

Estrogen secretion gradually decreases in women after menopause which leads to metabolic disorder, increased blood viscosity, and increased lipids, contributing to an atherosclerotic process and increasing the incidence of myocardial infarction [81]. Therefore, postmenopausal women were at risk of ACS, which were consistent with the included studies, which showed a higher proportion of post-menopausal patients in the ACS group [13, 15, 82]. However, the population of the target in this review is young women who are less likely to be in postmenopausal period, even though there were few women who had early menopause [13, 15].

## 5 LIMITATIONS

The main limitation of this systematic review and meta-analysis was the retrospective design of the included studies which are prone to selection and recall biases, even though a thorough and detailed data collection minimized these biases. Due to the retrospective nature of the included studies, there was no data available to determine the effect of exposure duration on the occurrence of ACS. The risk factors assessed in each study were different, thus a few risk factors were not included in the meta-analysis. There were some differences in the age cut-off and the operationalizing definition of each risk factor between studies which might affect the comparability between studies. Further studies are required to investigate and improve the limitations of this study.

## CONCLUSION

The risk of ACS in young women is multifactorial with a complex interrelationship. In this study, we found that diabetes mellitus, obesity and high BMI, hypercholesterolemia, smoking, hypertension, and family history are major risk factors for ACS in young women. The independent risk factors which are strongly related to ACS in young women were diabetes mellitus, hypertension, and hypercholesterolemia with odds ratios of 6.21, 5.32, and 4.07. Other risk factors which may be associated with an increased risk of ACS in young women are heavy alcohol consumption, oral contraceptive use, and postmenopausal state. Health promotion and effective intervention in this specific population regarding these risk factors can decrease young female cardiovascular morbidity and mortality as well as improve the quality of life of women.

## Figures and Tables

**Table 1:** Study and patient characteristics of the included studies.

**S. No.**	**Study (Year)**	**Study Design**	**Settings**	**Study Interval**	**Type of ACS**	**Age Cut-off (Years)**	**Arm**	**No. of Patients**	**Age (Years)**	**Risk Factors Assessed**
1	Beckowski *et al.* (2018)	Case control	Multicenter Registry (Polish Registry of Acute Coronary Syndrome), WOBASZ, and NATPOL, Poland	2007 - 2014	UA, NSTEMI, STEMI	≤45	Case	1941	42 (39-44)	Major ACS risk factors (HT, obesity, hypercholesterolemia, DM, and smoking), family history of CAD, kidney disease, lung disease, ischemic stroke, PAD, anthroprometric
							Control	1170	42 (39-44)	
2	Friedlander (2001)	Case control	Washington State, USA	July 1991 - February 1995	Acute myocardial infarction	18-44	Case	107	39.5 ± 4.5	Race, education, marital status, DM, HT, hypercholesterolemia, current smoking, sedentary lifestyle, coffee, BMI, laboratory parameter, genetic
							Control	526	37.7 ± 5.3	
3	La Vecchia *et al.* (1987)	Case control	34 Hospitals in Northern Italia	January 1983 - December 1984	Acute myocardial infarction	<45	Case	52	N/A	Smoking, DM, HT, HT in pregnancy, hyperlipidemia, obesity, parity, age at first birth, menopausal status, coffee, alcohol, OC, hormonal replacement treatment, family history of coronary heart disease, marital status, education, social class
							Control	91		
4	Lewis *et al.* (1997)	Case control	16 centers in Germany, the United Kingdom, France, Austria, and Switzerland	August 1993 - June 1996	Acute myocardial infarction	16-44	Case	182	N/A	Country, age, smoking, HT, aspirin, DM, high lipids, family history of MI, high alcohol, parity, current OC, first user of OC, BMI, preeclampsia history
							Control	635		
5	Liu *et al.* (2020)	Case control	Beijing Anzhen Hospital, China	January 2010 - August 2016	UA, NSTEMI, STEMI	19-44	Case	415	40.77 ± 4.02	Overweight, HT, hyperlipidemia, DM, depression or anxiety, gynecological disease, hyperuricemia, family history of CHD, hyperhomocysteinemia, hypothyroidism, hypercholesterolemia, high CRP, anemia, cardiac insufficiency, smoking, history of PCI, autoimmune disease, postmenopausal, OC, renal insufficiency, renal artery stenosis
6	Oliveira *et al.* (2007)	Case control	Department of Cardiology in 4 Hospitals in Porto, Portugal	2001 - 2003	NSTEMI, STEMI	18-45	Case	42	40.7 ± 3.4	Age, education, BMI, waist circumference, leisure-time physical activity, total energy intake, ethanol, caffeine, family history of CAD, angina, dyslipidemia, HT, DM, smoking
							Control	486	35.0 ± 7.7	
7	Tanis *et al.* (2003)	Case control	8 University Hospitals and 8 General Hospitals in Netherlands	January 1990 - October 1995	Acute myocardial infarction	18-49	Case	217	42.8 ± 6.1	Age, obese, caucasian ethnicity, OC, education level, HT, hypercholesterolemia, DM, smoking, family history of cardiovascular disease
							Control	763	38.7 ± 8.0	

**Abbreviations:** ACS, acute coronary syndrome; BMI, body mass index; CAD, coronary artery disease; CRP, C-reactive protein; DM, diabetes mellitus; HT, hypertension, N/A, not available; NSTEMI, nonST-elevation myocardial infarction; OC, oral contraceptive; PAD, peripheral artery disease; PCI, primary coronary intervention; STEMI, ST-elevation myocardial infarction; UA, unstable angina.

**Table 2:** Risk factors assessed in the included studies.

**S. No.**	**Study (Year)**	**Arm**	**No. of pts**	**BMI (kg/m2)**	**Obesity**	**Hyper-** **Cholesterolemia**	**HT**	**DM**	**Smoking**	**Family History of CAD**	**Race (Caucasian)**	**Alcohol**	**Coffee/** **Caffeine**	**OC**	**Parity**	**Marital Status**	**Post-menopause**
1	Beckowski *et al.* (2018)	Case	1941	26.7 ± 5.3	433	700	966	203	944	339	N/A	N/A	N/A	N/A	N/A	N/A	N/A
		Control	1170	25.4 ± 4.8	182	141	189	21	457	433							
2	Friedlander *et al.* (2001)	Case	107	29.3 ± 7.4	N/A	43	34	17	74	N/A	91	N/A	4.8 ± 5.4 cups/d	N/A	N/A	70	N/A N/A
		Control	526	24.8 ± 5.4		77	50	14	110	N/A	461		2.1 ± 3.0 cups/d			386	
3	La Vecchia *et al.* (1987)	Case	52	<25: 36, >25: 16	16	5	14	5	40	26	N/A	Drinks/d <3: 19, ≥3: 10	Cups/d <4: 30, ≥4: 18	Current: 3, Past: 15	1-2: 33, ≥3: 8	36	7
		Control	91	<25: 70, >25: 21	21	3	5	2	39	42		Drinks/d <3: 46, ≥3: 6	Cups/d <4: 58, ≥4: 23	Current: 6, Past: 12	1-2: 37, ≥3: 24	61	7
4	Lewis *et al.* (1997)	Case	182	≤20: 17, 21-25: 64, 26-30: 51, >30: 42	42	20	42	14	146	39	N/A	35	N/A	Current: 57, First: 8	167	N/A	N/A
		Control	635	≤20: 97, 21-25: 329, 26-30: 131, >30: 75	75	14	50	12	233	40		152		Current: 156, First: 16	526		
5	Liu *et al.* (2020)	Case	415	<24: 150, 24-26: 141, 27-29: 73, 30-39: 49, ≥40: 2	51	35	207	97	27	62	N/A	N/A	N/A	15	N/A	N/A	18
		Control	415	<24: 346, 24-26: 36, 27-29: 22, 30-39: 10, ≥40: 1	11	7	46	17	12	9				2			4
6	Oliveira *et al.* (2007)	Case	42	27.8 ± 5.7	N/A	13	16	4	28	12	Portuguese Caucasian	9.4 ± 15.3 g/d	96.6 ± 71.2 mg/d	N/A	N/A	N/A	N/A
		Control	486	24.9 ± 4.8		81	42	3	241	66		5.0 ± 9.3 g/d	80 ± 54.5 mg/d				
7	Tanis *et al.* (2003)	Case	217	25.5 ± 5.0	64	23	55	11	180	141	206	N/A	N/A	85	N/A	N/A	N/A
		Control	763	23.4 ± 3.7	90	22	47	10	318	262	719			276			

**Abbreviations:** BMI, body mass index; CAD, coronary artery disease; DM, diabetes mellitus; HT, hypertension; N/A, not available; OC, oral contraceptive; d, day; pts, patients.

## Data Availability

The data that support the findings of this study are available in the article.

## LIST OF ABBREVIATIONS

ACS: Acute Coronary Syndrome
NSTEMI: Non-St-Segmen Elevation Myocardial Infarction
PRISMA: Preferred Reporting Items for Systematic Review and Meta-Analysis
UA: Unstable Angina

## SUPPLEMENTARY MATERIAL

PRISMA checklist is available on the publisher’s website along with the published article.
