# Supplementary material for: The Risk Factors of Acute Coronary Syndrome in Young Women: A Systematic Review and Meta-Analysis
Source: Curr Cardiol Rev. 2023 Mar 22;19(3):E161122210969. doi: 10.2174/1573403X19666221116113208 (PMC10280997; doi:10.2174/1573403X19666221116113208)
Supplement: Supplementary file 1 — PRISMA checklist is available on the publisher’s website along with the published article. [file CCR-19-E161122210969_SD1.pdf]

Supplementary Material

The Risk Factors of Acute Coronary Syndrome in Young Women: A Systematic Review and Meta-Analysis

Sisca Natalia Siagian[1], Christianto Christianto[2,*], Phoniex Angellia[2] and Ho Indra Holiyono[2]

[1]Department of Cardiology and Vascular Medicine, Pediatric Cardiology and Congenital Heart Defect Division,, National Cardiovascular Center Harapan Kita, Universitas Indonesia, Jakarta, Indonesia; [2]Faculty of Medicine, Universitas Indonesia, Jakarta, Indonesia

Section and Topic	Item #	Checklist item	Location where item is reported
TITLE			
Title	1	Identify the report as a systematic review.	Page 1
ABSTRACT			
Abstract	2	See the PRISMA 2020 for Abstracts checklist.	Page 1
INTRODUCTION			
Rationale	3	Describe the rationale for the review in the context of existing knowledge.	Page 1: Line 3 (Left column), Line 6 (Right column)
Objectives	4	Provide an explicit statement of the objective(s) or question(s) the review addresses.	Page 1: Line 8 (Right column)
METHODS			
Eligibility criteria	5	Specify the inclusion and exclusion criteria for the review and how studies were grouped for the syntheses.	Page 2: Line 18 (Left column)
Information sources	6	Specify all databases, registers, websites, organisations, reference lists and other sources searched or consulted to identify studies. Specify the date when each source was last searched or consulted.	Page 2: Line 5 (Left column)
Search strategy	7	Present the full search strategies for all databases, registers and websites, including any filters and limits used.	Page 2: Line 12 (Left column)
Selection process	8	Specify the methods used to decide whether a study met the inclusion criteria of the review, including how many reviewers screened each record and each report retrieved, whether they worked independently, and if applicable, details of automation tools used in the process.	Page 2: Line 13 and 48 (Left column)
Data collection process	9	Specify the methods used to collect data from reports, including how many reviewers collected data from each report, whether they worked independently, any processes for obtaining or confirming data from study investigators, and if applicable, details of automation tools used in the process.	Page 2: Line 33 (Left column)
Data items	10a	List and define all outcomes for which data were sought. Specify whether all results that were compatible with each outcome domain in each study were sought (e.g. for all measures, time points, analyses), and if not, the methods used to decide which results to collect.	Page 2: Line 34 (Left column)
	10b	List and define all other variables for which data were sought (e.g. participant and intervention characteristics, funding sources). Describe any assumptions made about any missing or unclear information.	Not available
Study risk of bias assessment	11	Specify the methods used to assess risk of bias in the included studies, including details of the tool(s) used, how many reviewers assessed each study and whether they worked independently, and if applicable, details of automation tools used in the process.	Page 2: Line 48 (Left column)<br>Page 6: Line 30

Section and Topic	Item #	Checklist item	Location where item is reported
			(Left column)
Effect measures	12	Specify for each outcome the effect measure(s) (e.g. risk ratio, mean difference) used in the synthesis or presentation of results.	Page 2: Line 53 (Left column)
Synthesis methods	13a	Describe the processes used to decide which studies were eligible for each synthesis (e.g. tabulating the study intervention characteristics and comparing against the planned groups for each synthesis (item #5)).	Page 2: Line 16 (Right column)
	13b	Describe any methods required to prepare the data for presentation or synthesis, such as handling of missing summary statistics, or data conversions.	Page 2: Line 53 (Left column)
	13c	Describe any methods used to tabulate or visually display results of individual studies and syntheses.	Page 2: Line 34 and 53 (Left column)
	13d	Describe any methods used to synthesize results and provide a rationale for the choice(s). If meta-analysis was performed, describe the model(s), method(s) to identify the presence and extent of statistical heterogeneity, and software package(s) used.	Page 2: Line 4 (Right column)
	13e	Describe any methods used to explore possible causes of heterogeneity among study results (e.g. subgroup analysis, meta-regression).	Not available
	13f	Describe any sensitivity analyses conducted to assess robustness of the synthesized results.	Not available
Reporting bias assessment	14	Describe any methods used to assess risk of bias due to missing results in a synthesis (arising from reporting biases).	Page 2: Line 44 (Left column)
Certainty assessment	15	Describe any methods used to assess certainty (or confidence) in the body of evidence for an outcome.	Page 11-12
RESULTS			
Study selection	16a	Describe the results of the search and selection process, from the number of records identified in the search to the number of studies included in the review, ideally using a flow diagram.	Page 2: Line 13 (Right column)
	16b	Cite studies that might appear to meet the inclusion criteria, but which were excluded, and explain why they were excluded.	Not available
Study characteristics	17	Cite each included study and present its characteristics.	Page 2: Line 27 (Right column)
Risk of bias in studies	18	Present assessments of risk of bias for each included study.	Page 2: Line 48 (Left column)
Results of individual studies	19	For all outcomes, present, for each study: (a) summary statistics for each group (where appropriate) and (b) an effect estimate and its precision (e.g. confidence/credible interval), ideally using structured tables or plots.	Page 2: Line 56 (Right column), Page 11
Results of syntheses	20a	For each synthesis, briefly summarise the characteristics and risk of bias among contributing studies.	Page 2: Line 48 (Left column), Page 2: Line 56 (Right column), Page 11
	20b	Present results of all statistical syntheses conducted. If meta-analysis was done, present for each the summary estimate and its precision (e.g. confidence/credible interval) and measures of statistical heterogeneity. If comparing groups, describe the direction of the effect.	Page 11-12
	20c	Present results of all investigations of possible causes of heterogeneity among study results.	Not available
	20d	Present results of all sensitivity analyses conducted to assess the robustness of the synthesized results.	Not available
Reporting biases	21	Present assessments of risk of bias due to missing results (arising from reporting biases) for each synthesis assessed.	Page 10
Certainty of evidence	22	Present assessments of certainty (or confidence) in the body of evidence for each outcome assessed.	Page 11-12

Section and Topic	Item #	Checklist item	Location where item is reported
DISCUSSION			
Discussion	23a	Provide a general interpretation of the results in the context of other evidence.	Page 6: Line 43 and 46 (Left column)
	23b	Discuss any limitations of the evidence included in the review.	Page 6: Line 34 (Left column)
	23c	Discuss any limitations of the review processes used.	Page 6: Line 27 (Left column)
	23d	Discuss implications of the results for practice, policy, and future research.	Page 6: Line 53 (Left column)
OTHER INFORMATION			
Registration and protocol	24a	Provide registration information for the review, including register name and registration number, or state that the review was not registered.	Not available
	24b	Indicate where the review protocol can be accessed, or state that a protocol was not prepared.	Not available
	24c	Describe and explain any amendments to information provided at registration or in the protocol.	Not available
Support	25	Describe sources of financial or non-financial support for the review, and the role of the funders or sponsors in the review.	Page 6: Line 5 (Right column)
Competing inter-ests	26	Declare any competing interests of review authors.	Page 6: Line 11 (Right column)
Availability of data, code and other materials	27	Report which of the following are publicly available and where they can be found: template data collection forms; data extracted from included studies; data used for all analyses; analytic code; any other materials used in the review.	Page 6: Line 21 (Right column)

From: Page MJ, McKenzie JE, Bossuyt PM, Boutron I, Hoffmann TC, Mulrow CD, et al. The PRISMA 2020 statement: an updated guideline for reporting systematic reviews. BMJ 2021;372:n71. doi: 10.1136/bmj.n71
For more information, visit: http://www.prisma-statement.org/
